# Supplementary material for: Targeting nucleic acid phase transitions as a mechanism of action for antimicrobial peptides
Source: Nat Commun. 2023 Nov 7;14:7170. doi: 10.1038/s41467-023-42374-4 (PMC10630377; doi:10.1038/s41467-023-42374-4)
Supplement: Supplementary file 1 — Supplementary Information [file 41467_2023_42374_MOESM1_ESM.pdf]

Supplementary Information

Targeting Nucleic Acid Phase Transitions as a Mechanism of Action for Antimicrobial Peptides

Tomas Sneideris[1,‡], Nadia A. Erkamp[1,‡], Hannes Ausserwöger[1,‡], Kadi L. Saar[1],Timothy J. Welsh[1], Daoyuan Qian[1], Kai Katsuya Gaviria[2] Margaret L.L.Y. Johncock[1], Georg Krainer[1], Alexander Borodavka[2*] and Tuomas P. J. Knowles[1,3,*]

[1]Yusuf Hamied Department of Chemistry, University of Cambridge, Lensfield Road, Cambridge CB21EW, United Kingdom

[2]Department of Biochemistry, University of Cambridge, United Kingdom

[3]Cavendish Laboratory, Department of Physics, University of Cambridge, J J Thomson Ave, Cambridge, CB30HE, United Kingdom

‡These authors contributed equally to this work

*Correspondence: tpjk2@cam.ac.uk (T.P.J.K) or ab2677@cam.ac.uk (A.B.)

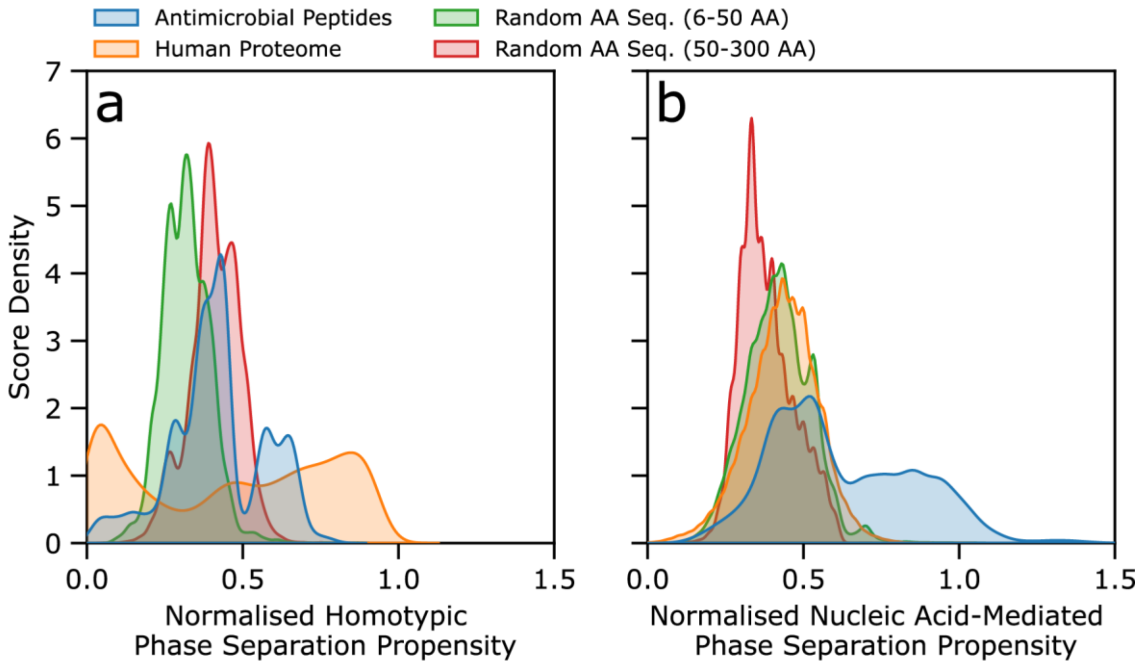

Supplementary Figure 1. Homotypic (**a**) and oligonucleotide-mediated (**b**) phase separation propensity density distribution plots for AMPs (n=13170), human proteins (n=20324), and random amino acid (AA) sequences (n=10000) of different lengths.

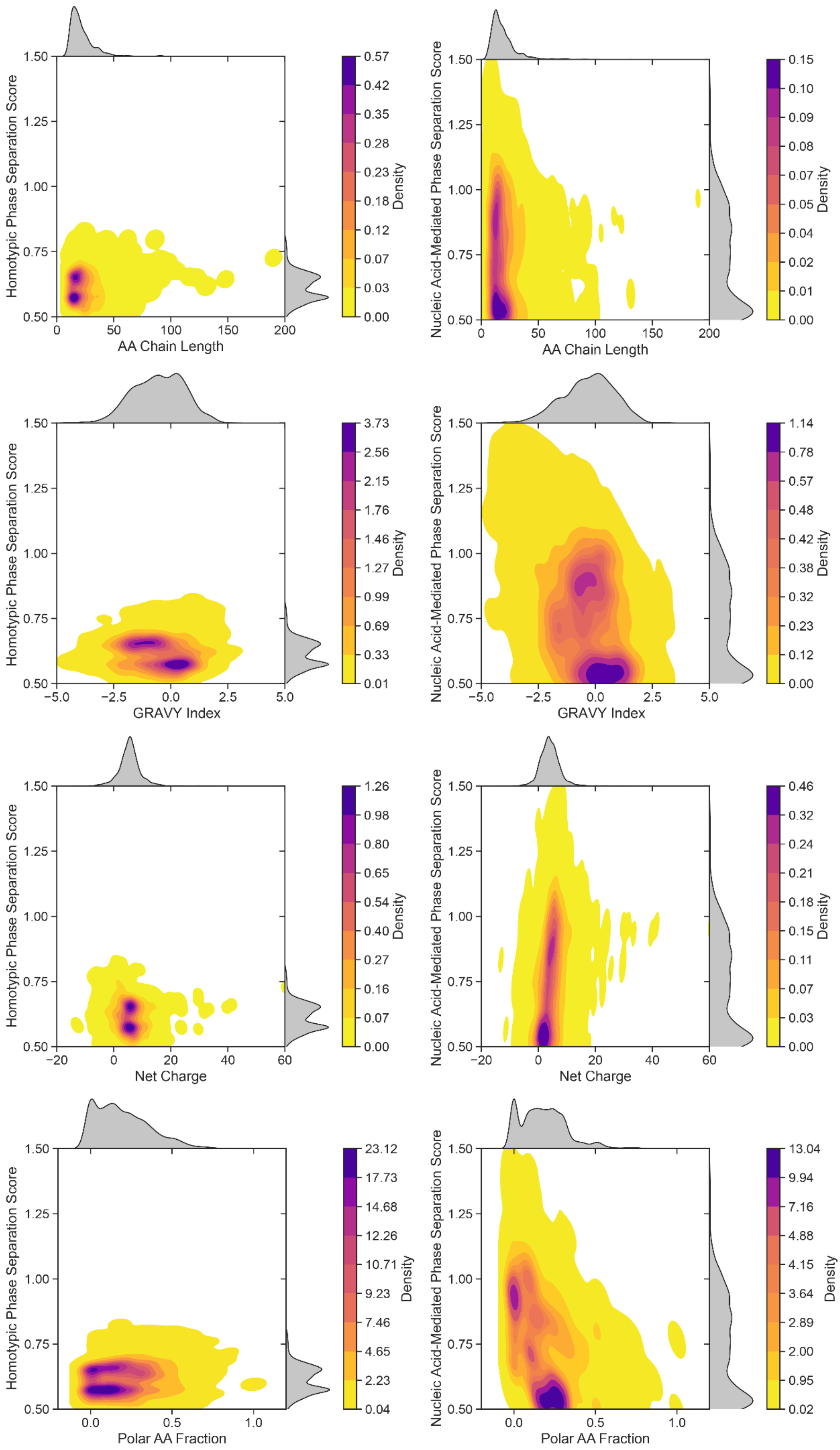

Supplementary Figure 2. Density plots of AMP phase separation score against AMP chain length, net charge, GRAVY index and polar amino acid fraction.

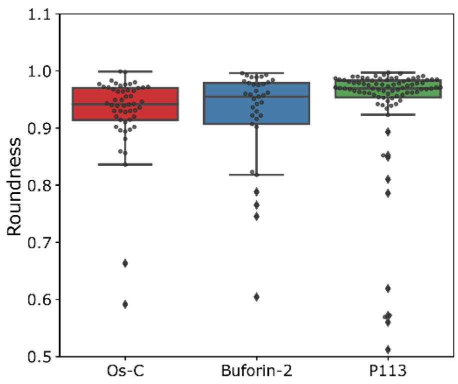

Supplementary Figure 3. Roundness of Os-C-, Buforin-2- and P113-Poly(A) RNA condensates. Roundness was calculated using the following formula: $roundness = \frac{4*\text{area}}{\pi*\text{major\_axis}^2}$. Image analysis was performed using Fiji/ImageJ software. $n_{\text{Os-C}} = 53$, $n_{\text{Buforin-2}} = 38$, $n_{\text{P113}} = 89$.

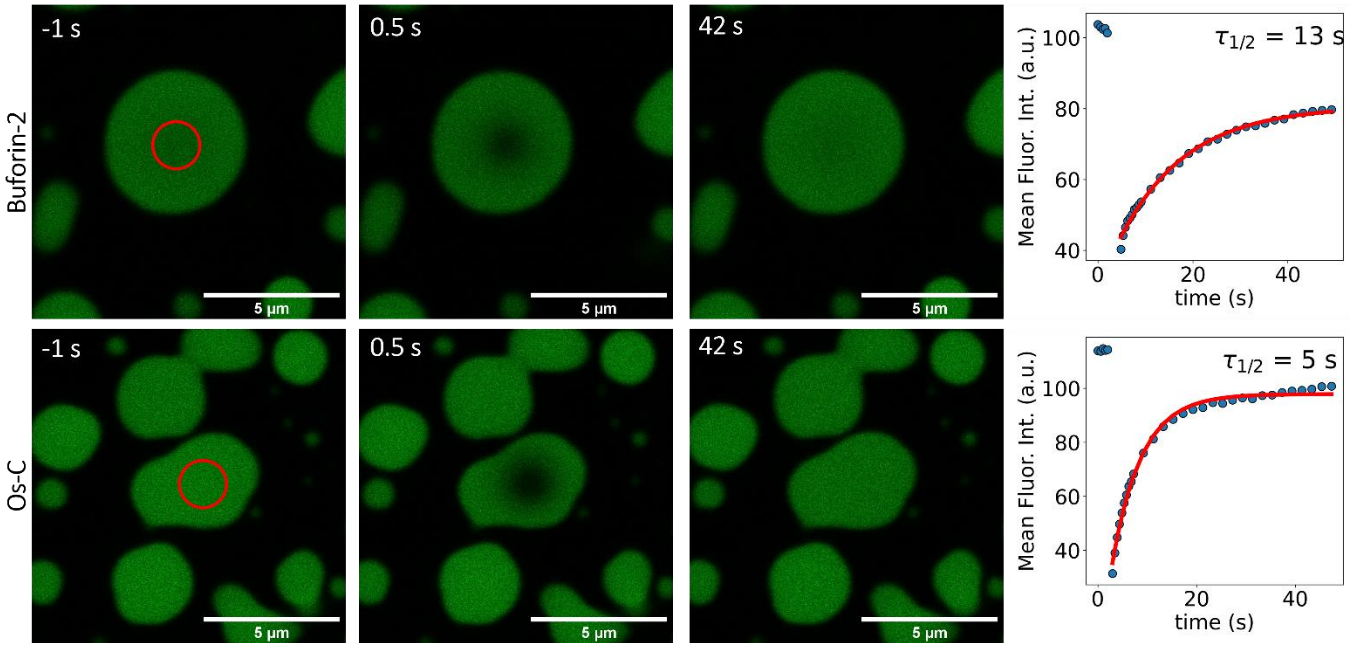

Supplementary Figure 4. Representative confocal microscopy images of FRAP analysis in Buforin-2- or Os-C-Poly(A) RNA condensates and the kymograph of the FRAP experiment (n = 4 biological replicates).

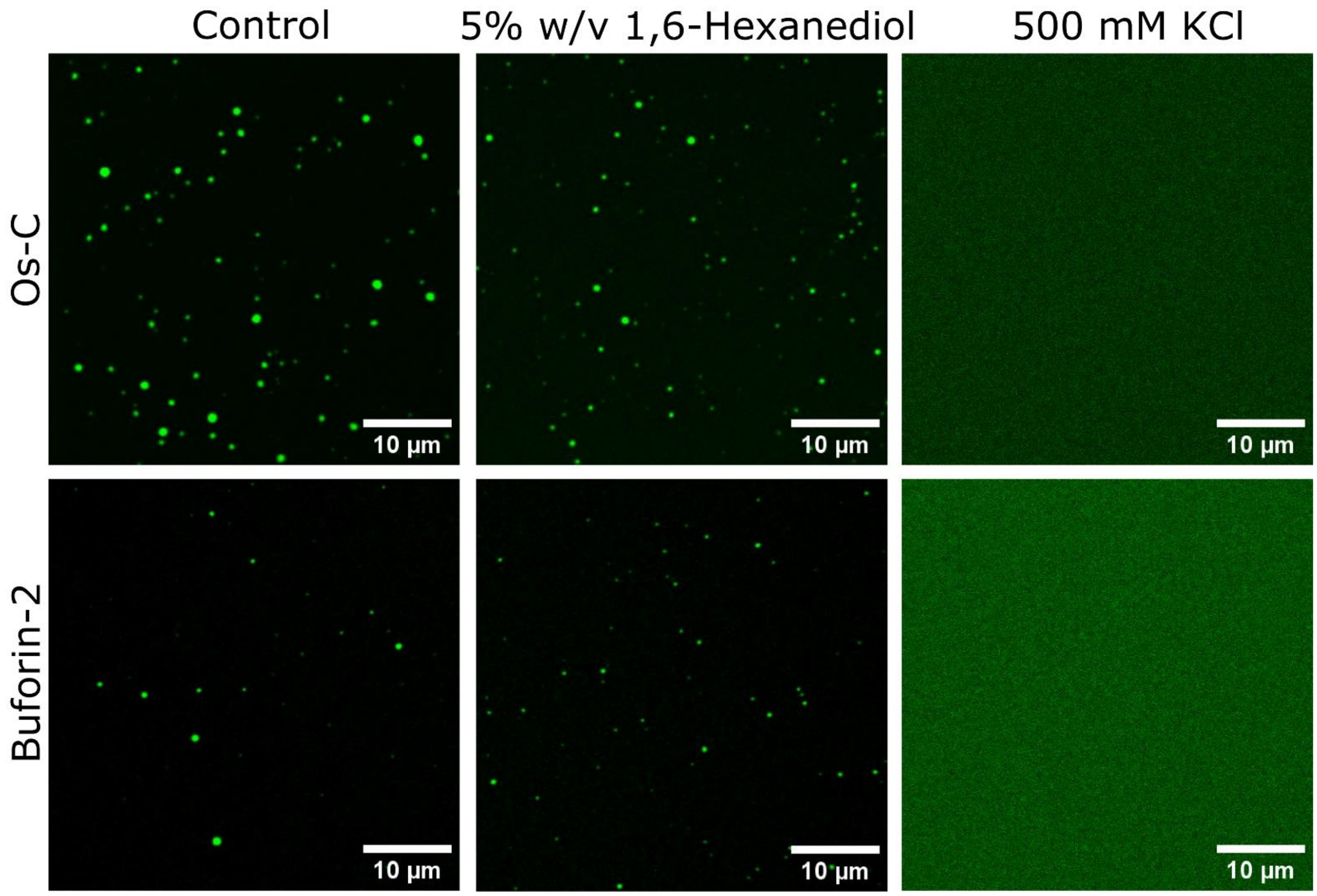

Supplementary Figure 5. Representative confocal fluorescence microscopy images of Os-C- and Buforin-2-Yeast RNA condensates before the introduction of 1,6-hexanediol or a high concentration of KCl. Experiments were repeated 3 times with similar results.

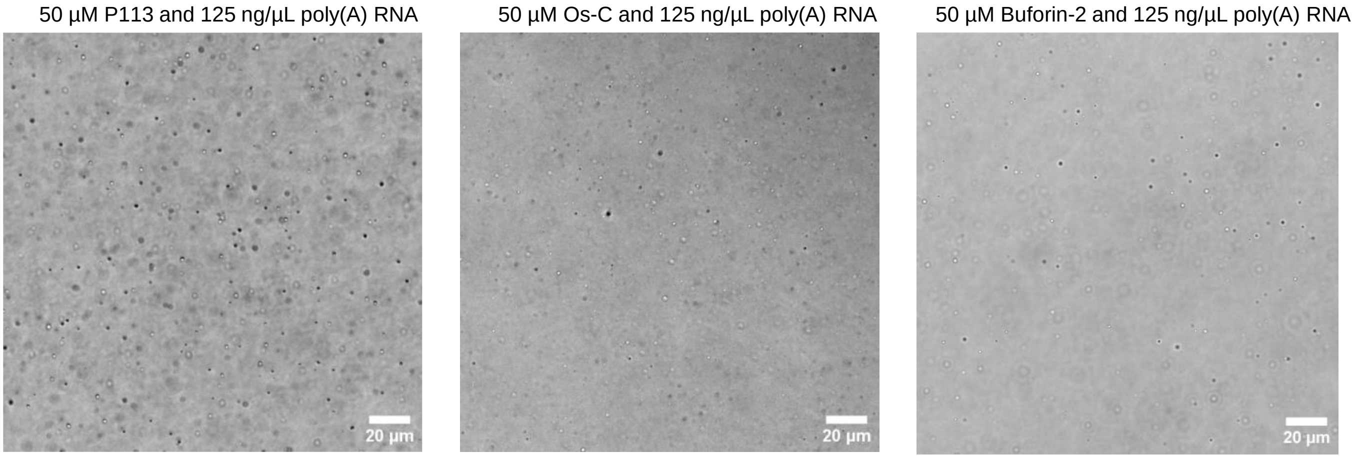

Supplementary Figure 6. Brightfield images of AMP-poly(A) RNA condensates formed in 10 mM sodium phosphate pH 7.3 supplemented with 150 mM NaF.

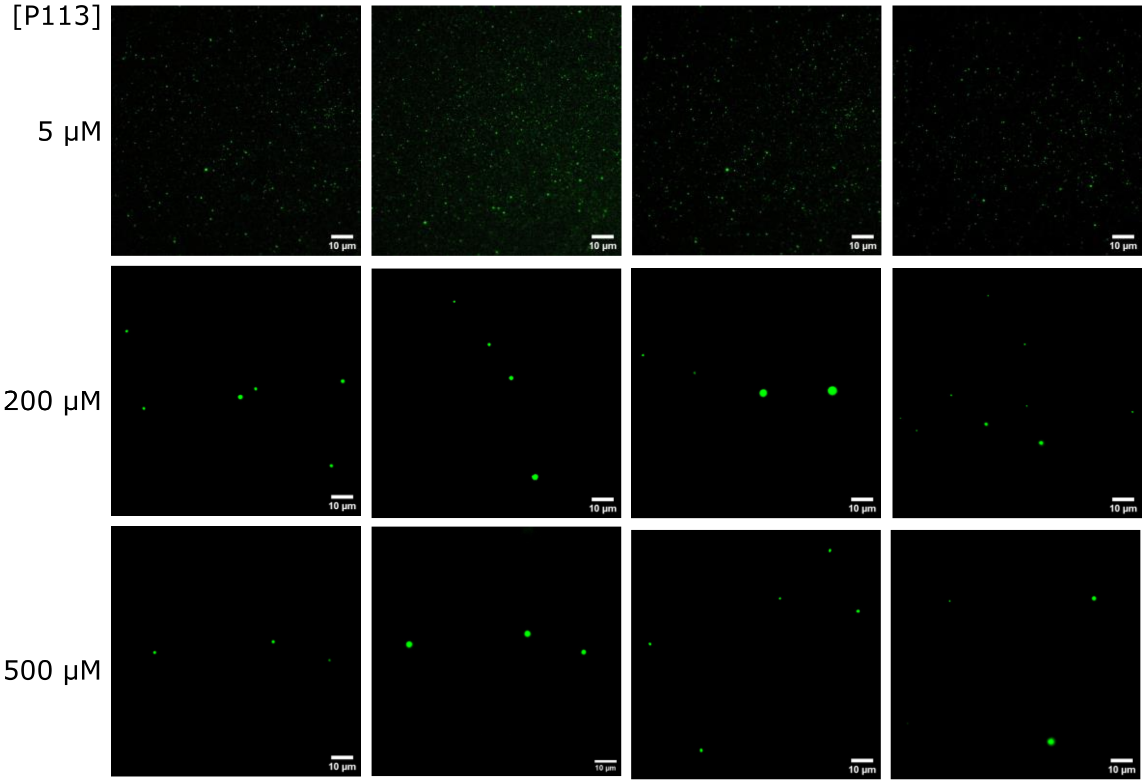

Supplementary Figure 7. Epifluorescence microscopy images of the Atto 647N-labelled 16S rRNA (0.2 nM final concentration) samples containing varying concentrations of the P113 peptide. Multiple regions of interest are shown for every sample. Experiments were repeated 3 times with similar results.

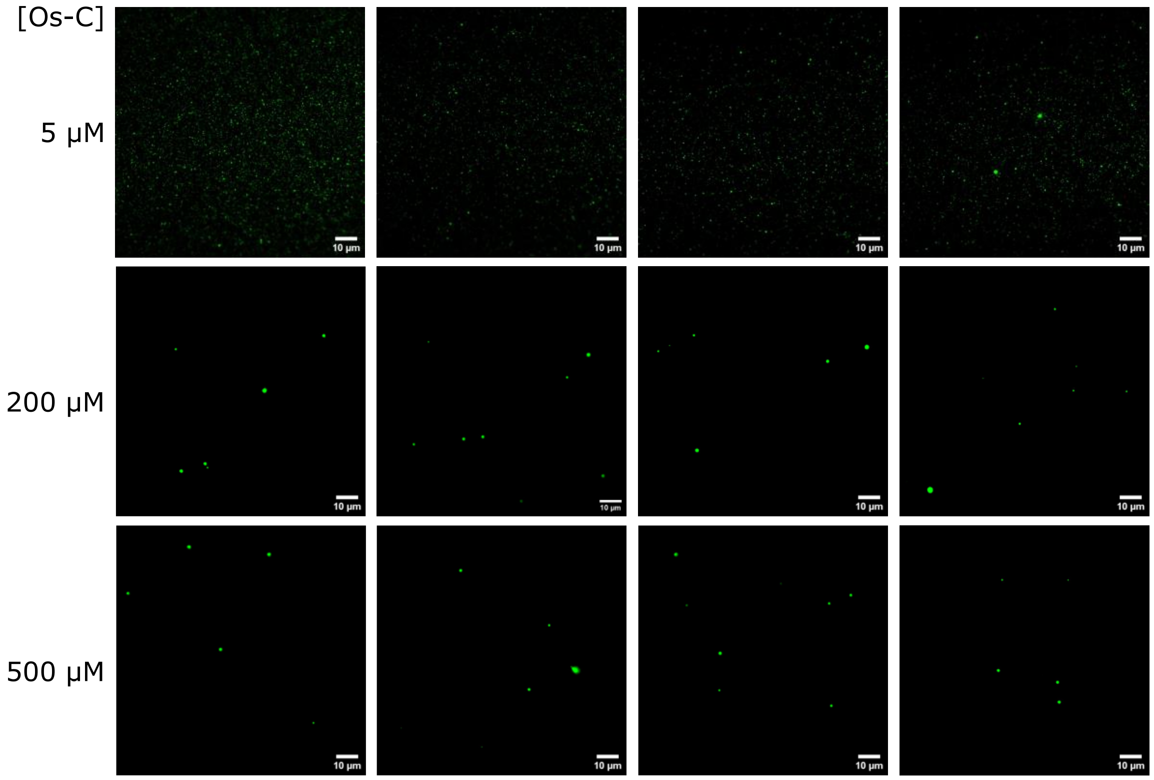

Supplementary Figure 8. Epifluorescence microscopy images of the Atto 647N-labelled 16S rRNA (0.2 nM final concentration) samples containing varying concentrations of the Os-C peptide. Multiple regions of interest are shown for every sample. Experiments were repeated 3 times with similar results.

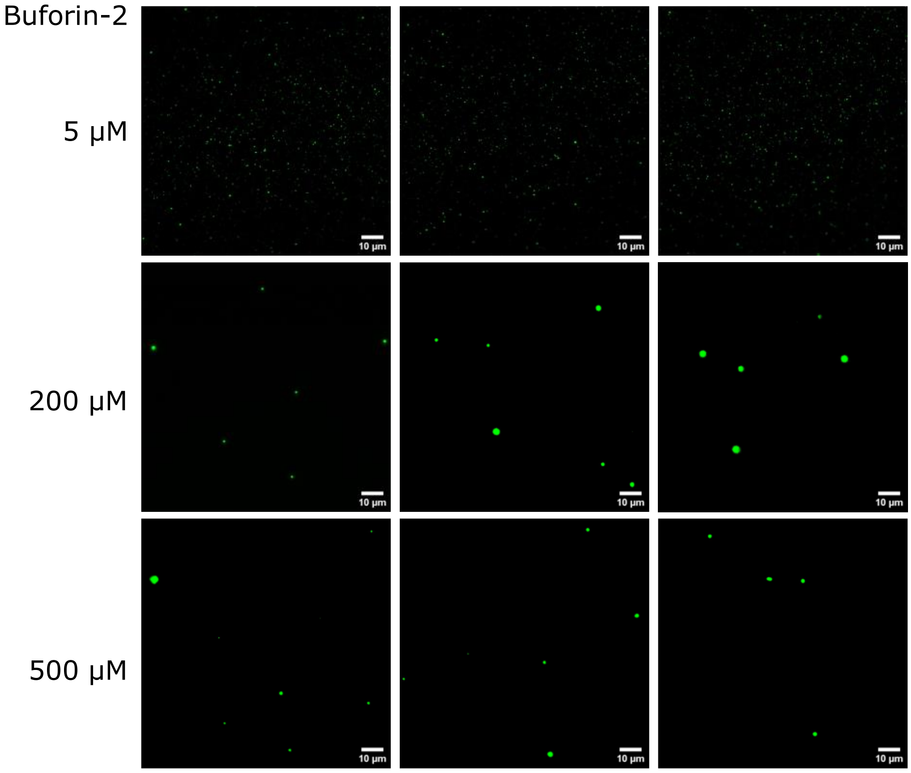

Supplementary Figure 9. Epifluorescence microscopy images of the Atto 647N-labelled 16S rRNA (0.2 nM final concentration) samples containing varying concentrations of Buforin-2. Multiple regions of interest are shown for every sample. Experiments were repeated 3 times with similar results.

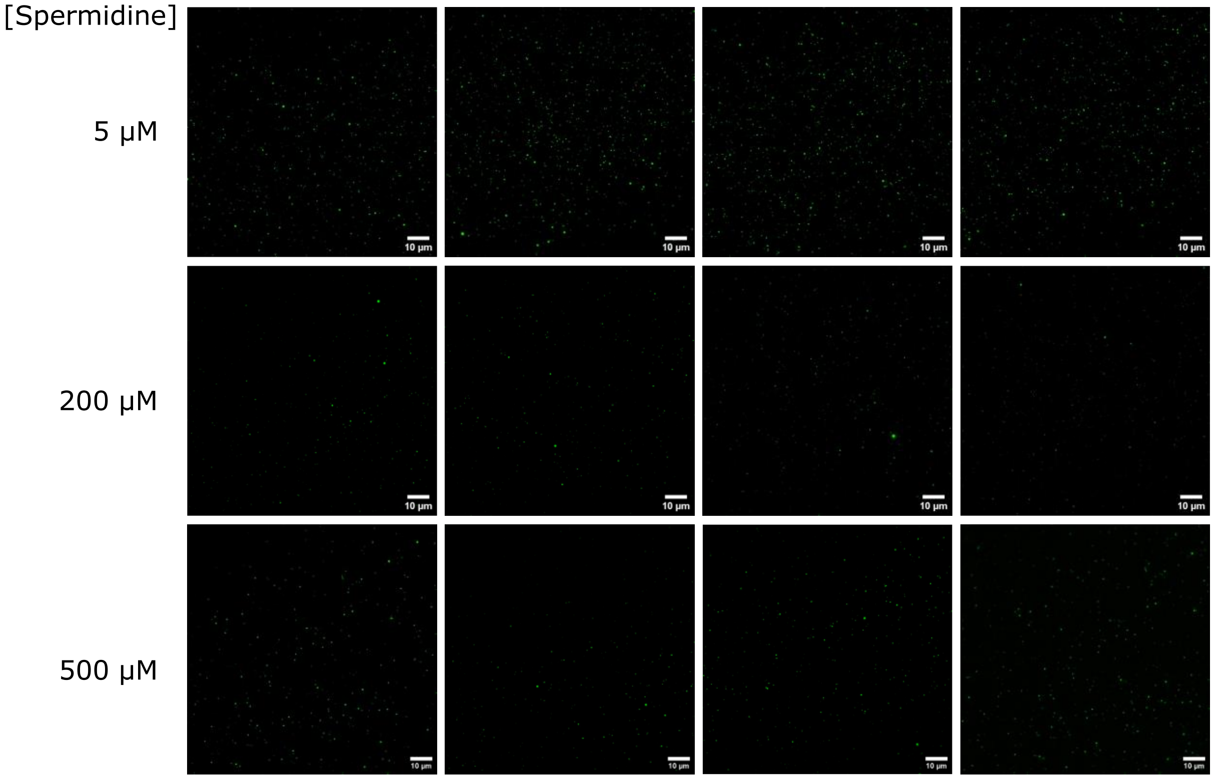

Supplementary Figure 10. Epifluorescence microscopy images of the Atto 647N-labelled 16S rRNA (0.2 nM final concentration) samples containing varying concentrations of spermidine. Multiple regions of interest are shown for every sample. Experiments were repeated 3 times with similar results.

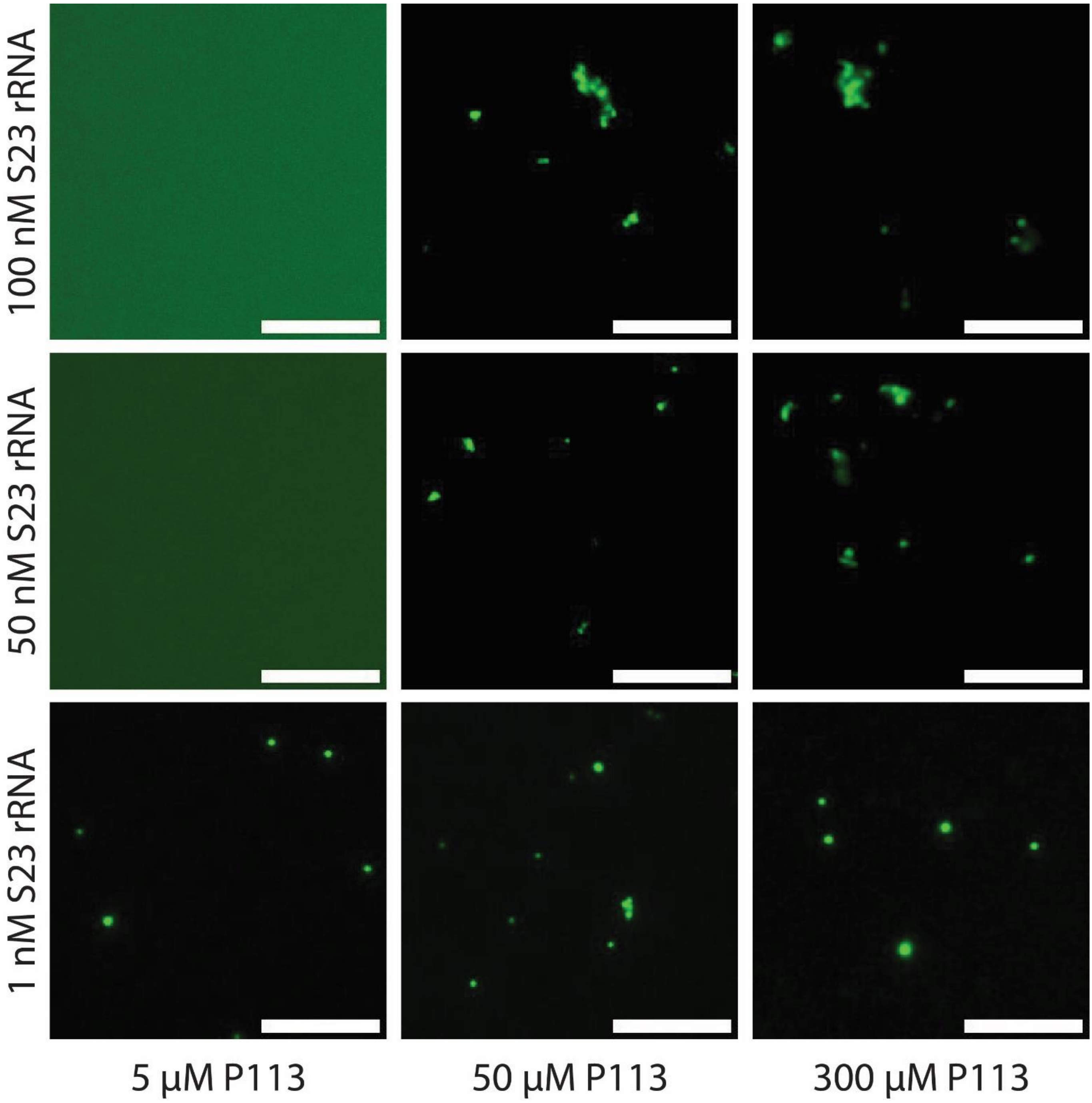

Supplementary Figure 11. Epifluorescence microscopy images of the Atto 647N-labelled 23S rRNA (1-100 nM final concentration) samples containing varying concentrations of P113. The scale bares are 10 µm. Experiments were repeated 2 times with similar results.

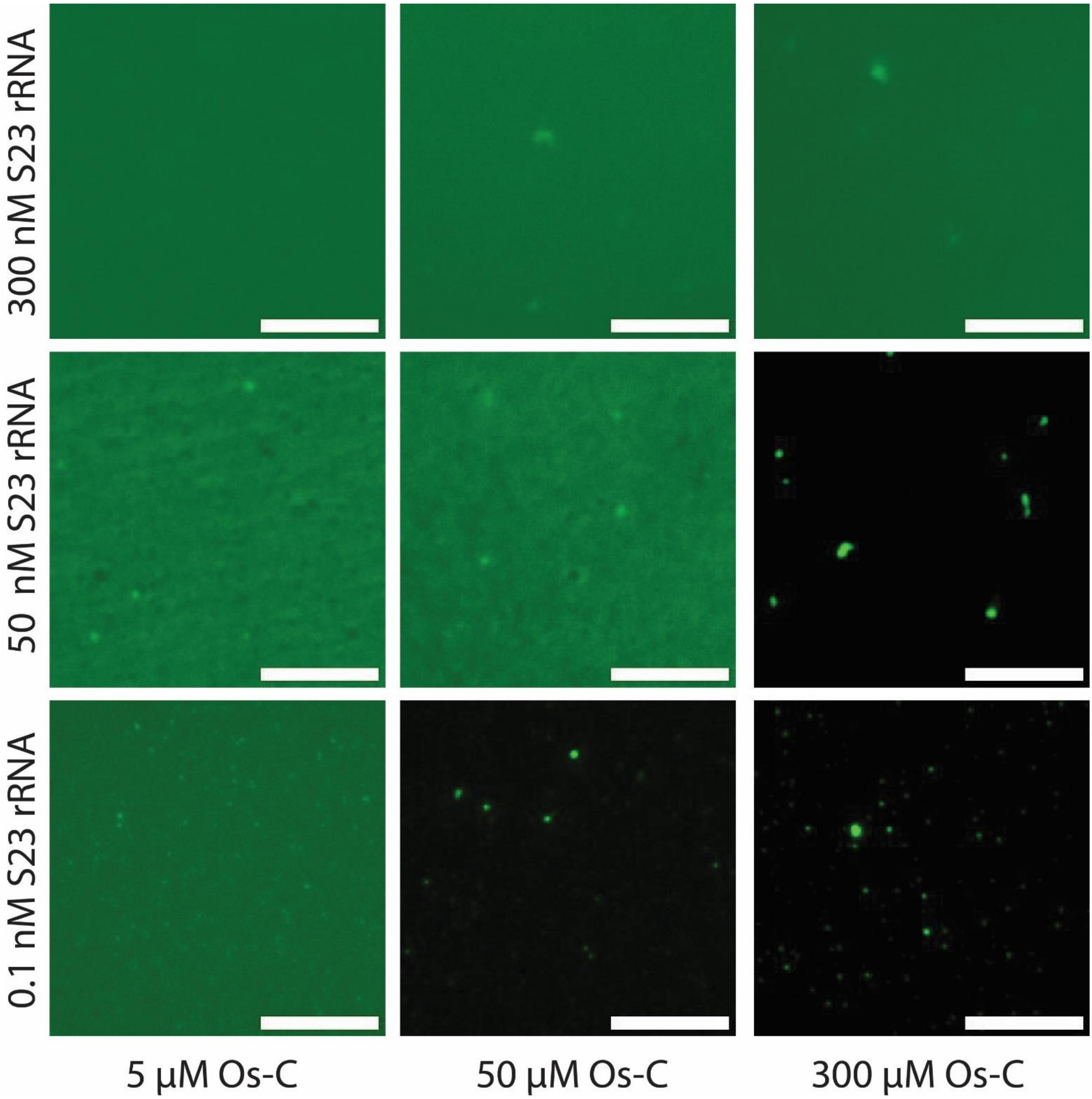

Supplementary Figure 12. Epifluorescence microscopy images of the Atto 647N-labelled 23S rRNA (0.1-300 nM final concentration) samples containing varying concentrations of Os-C. The scale bares are 10 µm. Experiments were repeated 2 times with similar results.

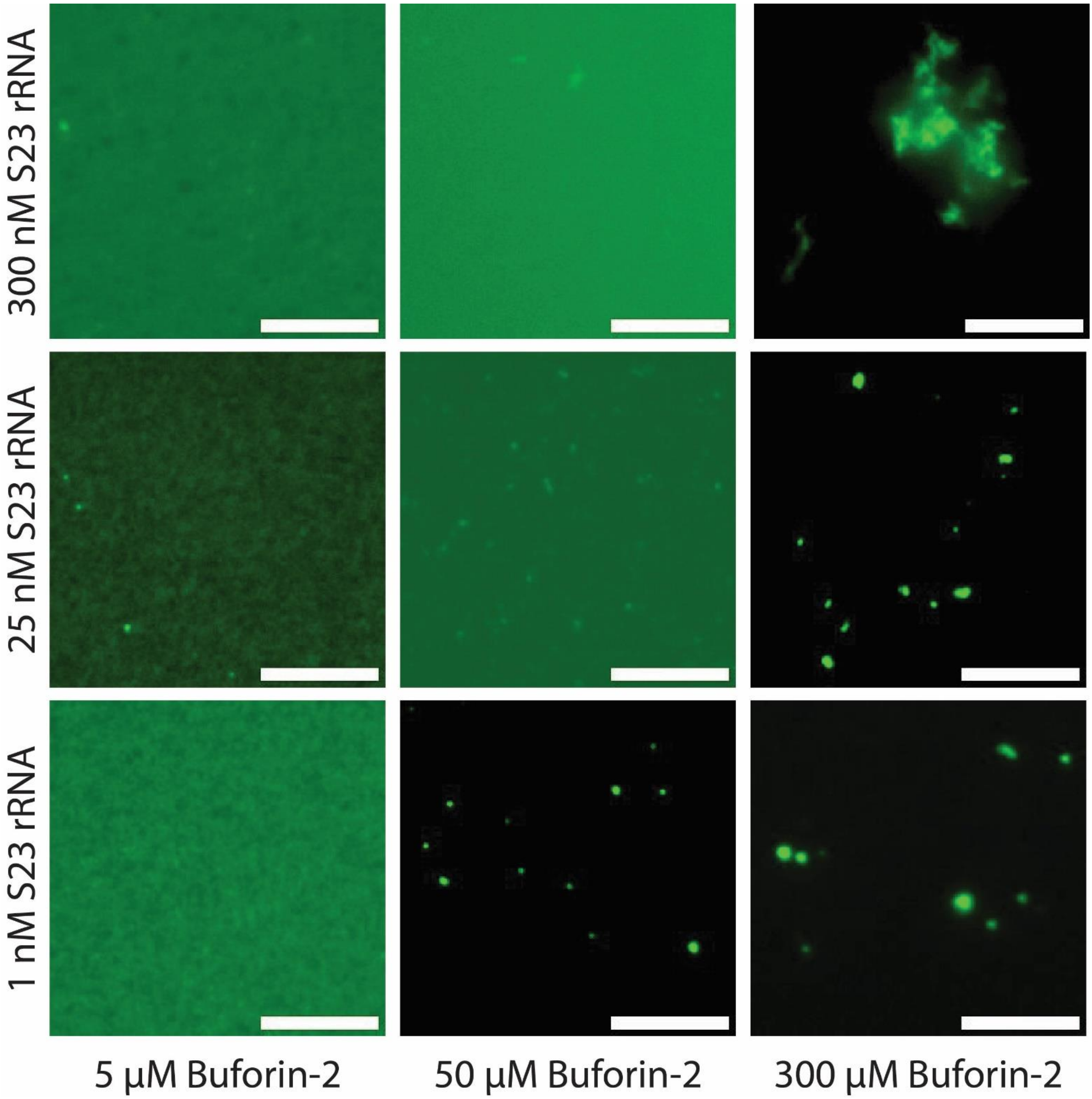

Supplementary Figure 13. Epifluorescence microscopy images of the Atto 647N-labelled 16S rRNA (1-300 nM final concentration) samples containing varying concentrations of Buforin-2. The scale bares are 10 µm. Experiments were repeated 2 times with similar results.

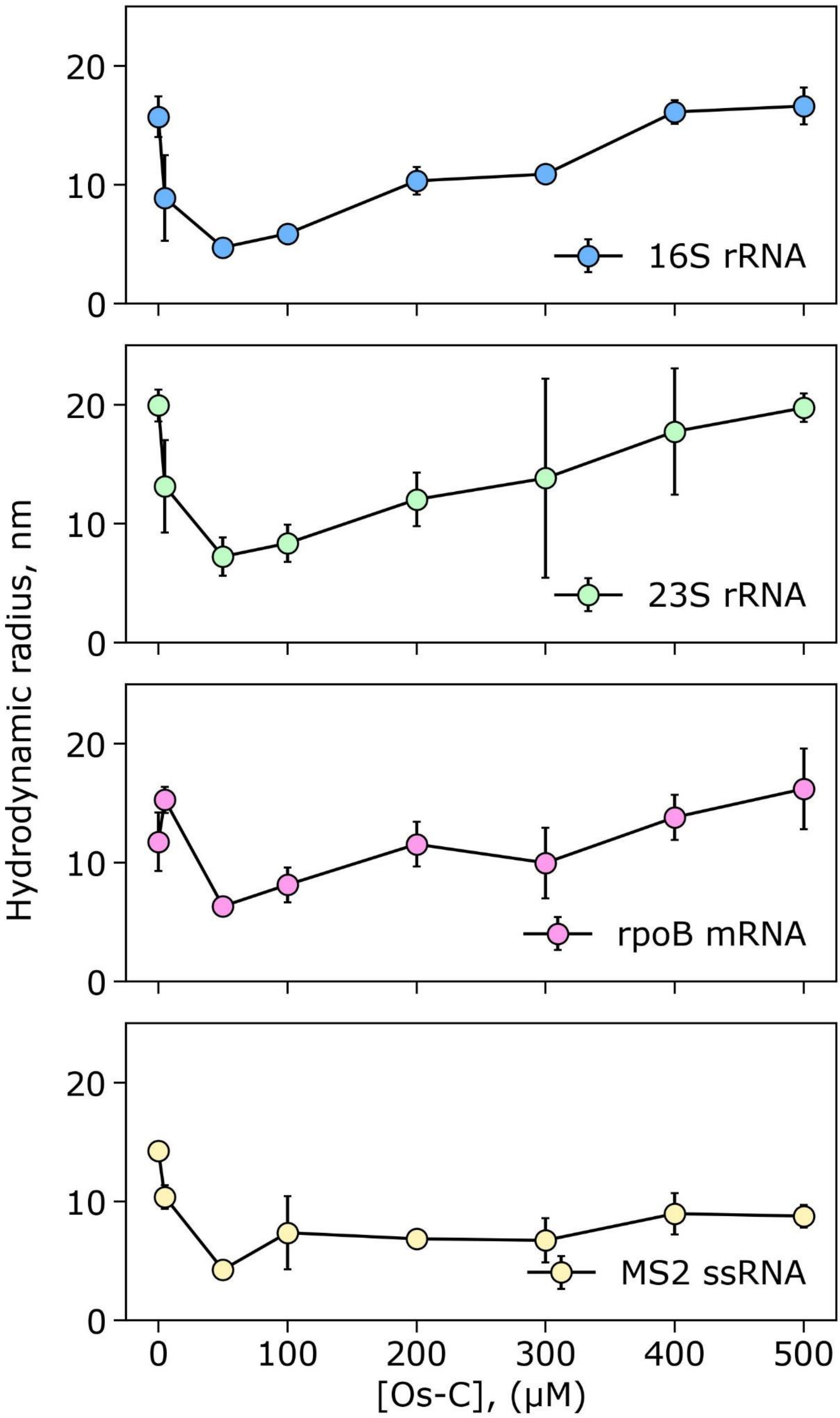

Supplementary Figure 14. MDS measurements of 16S rRNA, 23S rRNA, rpoB mRNA and MS2 ssRNA in the presence of Os-C antimicrobial peptide. Data are presented as mean values ± SD (n = 3 technical replicates).

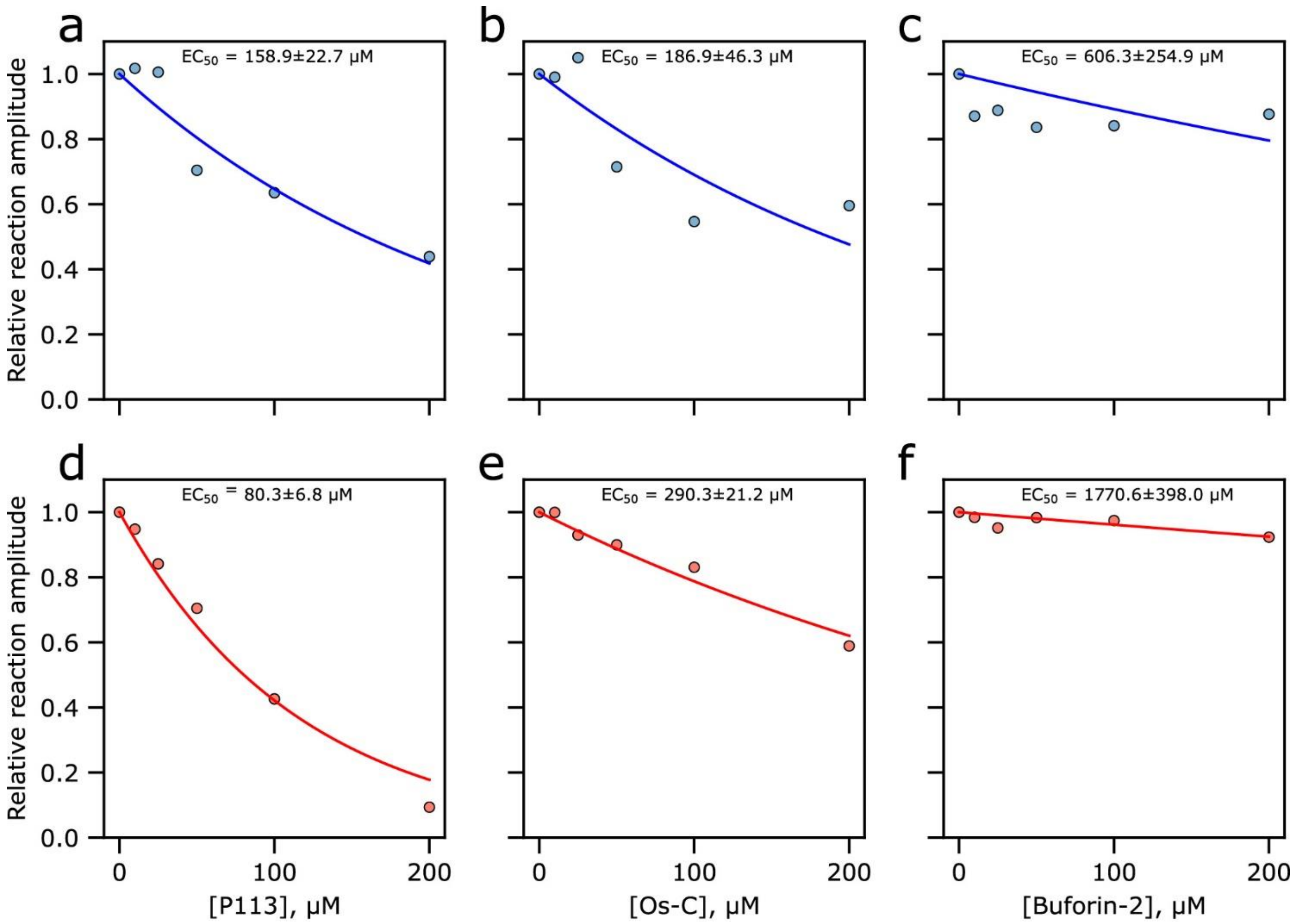

Supplementary Figure 15. Effect of AMPs on relative CFPS reaction aplitude. Relative reaction amplitudes of DNA template-initiated (**a-c**) or mRNA-initiated (**d-f**) reaction. The solid lines are the exponential fits: $y = 2^{\frac{-[AMP]}{EC_{50}}}$, where [AMP] is the concentration of antimicrobial peptide and EC_{50} is half maximal effective concentration. EC_{50} error is the RMS (root mean square).

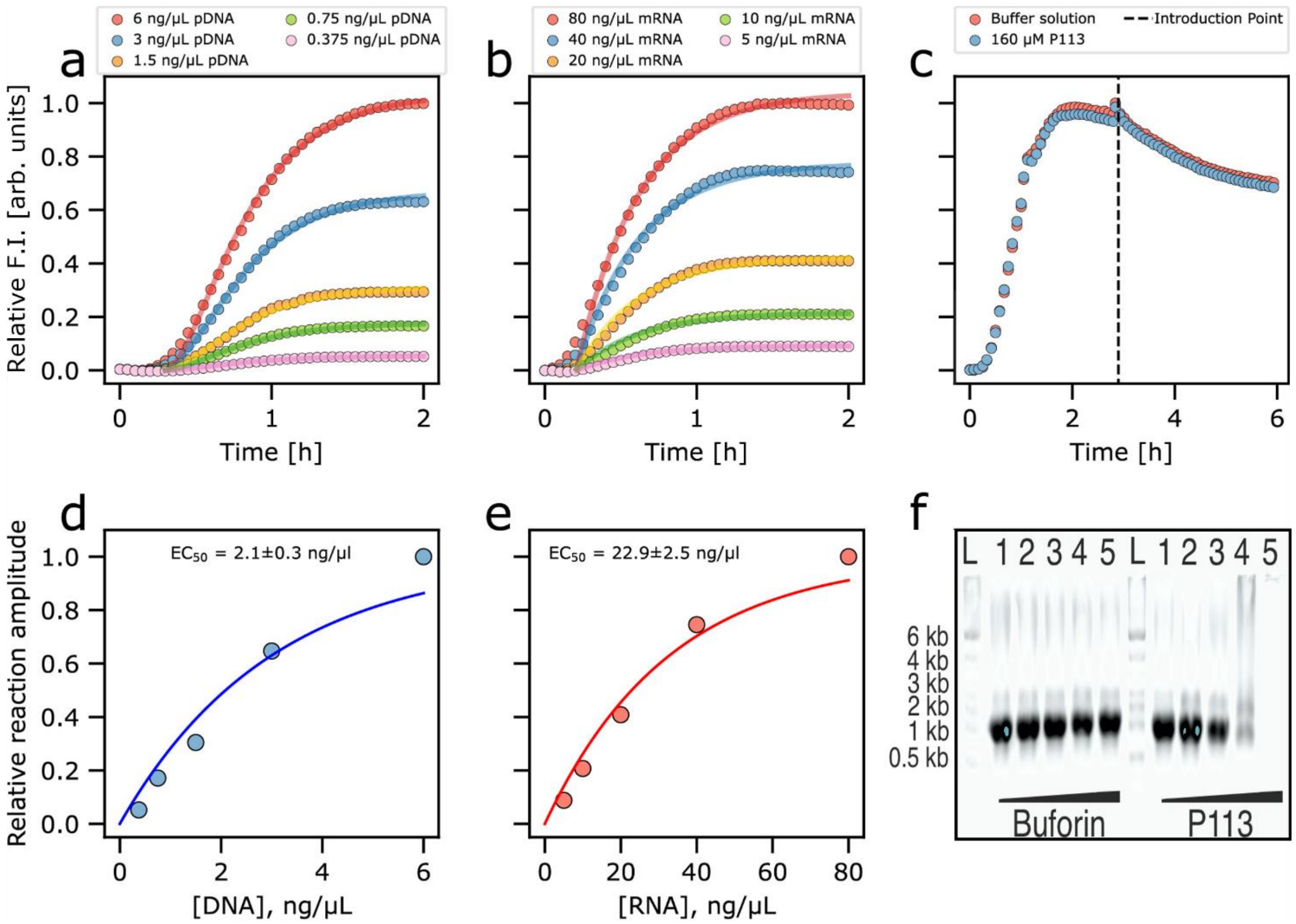

Supplementary Figure 16. Cell-free eGFP expression reaction initiated using plasmid DNA (**a, c**) or mRNA (**b**). Cell-free eGFP synthesis reaction where P113 peptide solution or buffer solution was introduced after 3h since the reaction started (**c**). Relative reaction amplitudes of DNA template-initiated (**d**) or mRNA-initiated (**e**) reaction. The solid lines are the exponential fits: $y = 1 - 2^{\frac{-[NA]}{EC_{50}}}$, where [NA] is the initial concentration of nucleic acid template and EC_{50} is half maximal effective concentration.In vitro transcription reaction in the presence of varying concentrations of Buforin-2 or P113 peptides (f). eGFP transcripts (~1 kb) were produced by adding 25 ng/µl of the template plasmid into T7 polymerase-driven in vitro transcription reaction in the presence of 50-300 µM each peptide. L denotes RNA size marker, lanes 1 – 0 µM peptide (buffer only); lanes 2-5 correspond to 50, 100, 200 and 300 µM peptide concentrations. Note that the additional peptide constituted less than 5% (v/v) of the total reaction volume at the highest concentration shown. P113 significantly inhibits the transcription reaction in the 200 µM range (lane 4).

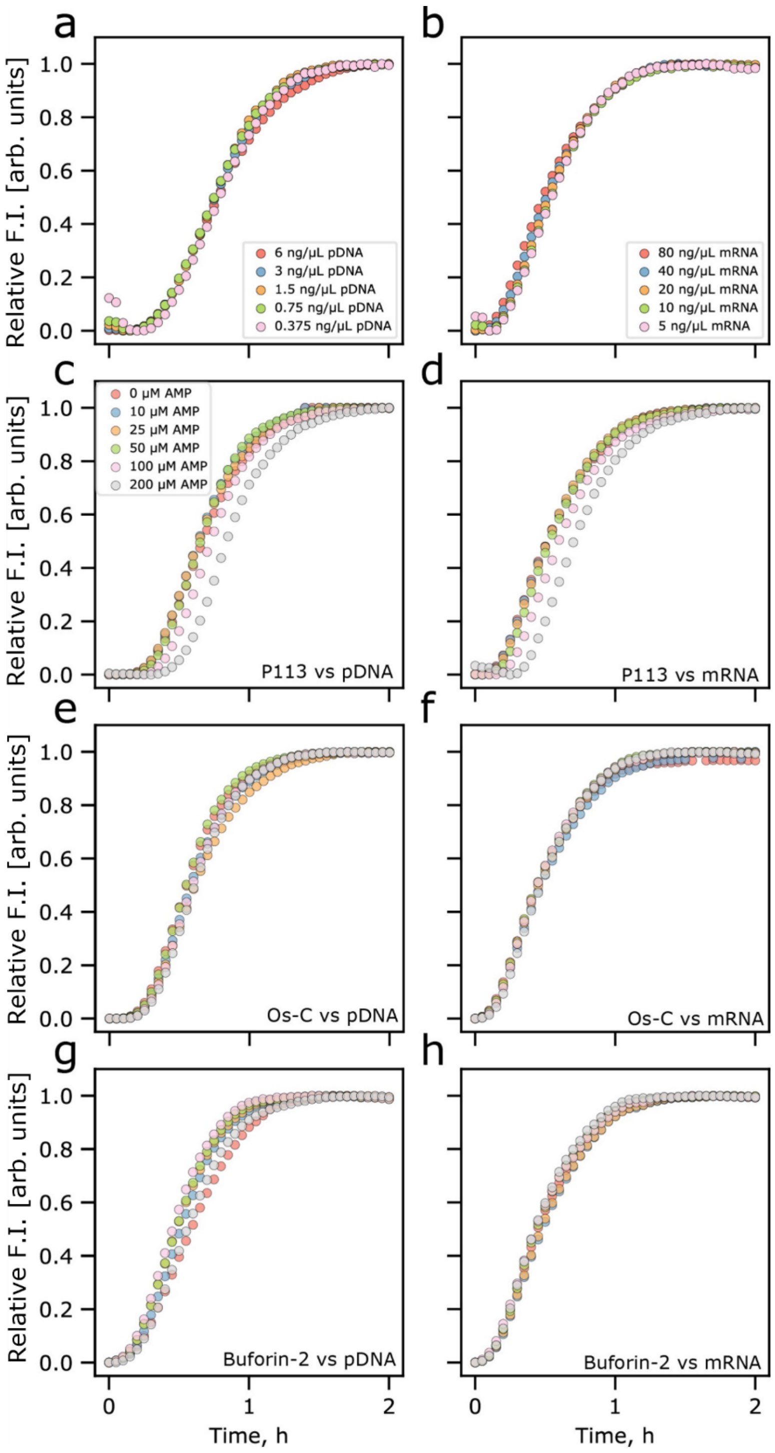

Supplementary Figure 17. Kinetics of cell-free eGFP protein synthesis. Normalised kinetic curves of CFPS reaction initiated by plasmid DNA (pDNA) (**a, c, e, g**) or mRNA (**b, d, f, h**) at varying pDNA (**a**), mRNA (**b**), or AMP (**c, d, e, f, g, h**) concentrations.

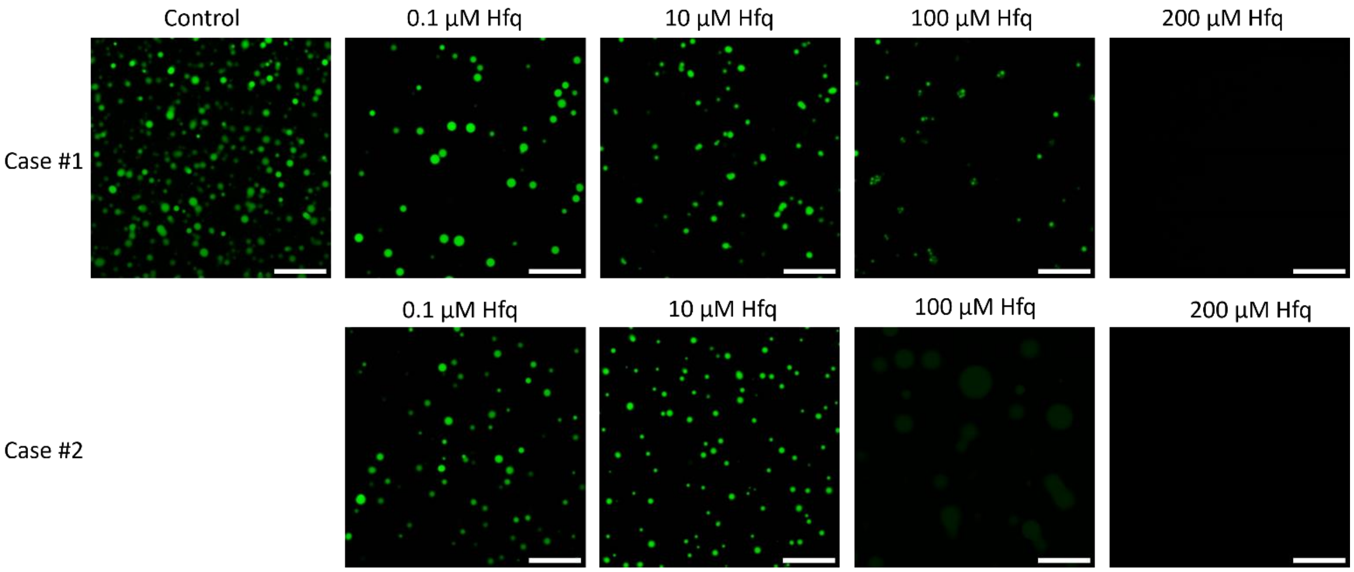

Supplementary Figure 18. Os-C vs. Hfq competition assay. Case #1: 300 µM Os-C was mixed with 250 ng/µL of poly(A) and incubated for 5 min at room temperature. Subsequently, 0.1-200 µM Hfq was introduced and the sample was incubated for an additional 5 min before imaging via a confocal fluorescence microscope. Case #2: 0.1-200 µM Hfq was pre-incubated with 250 ng/µL of poly(A) for 5 min before introducing 300 µM Os-C. Experiments were repeated 3 times with similar results. The scale bars are 20 µm.

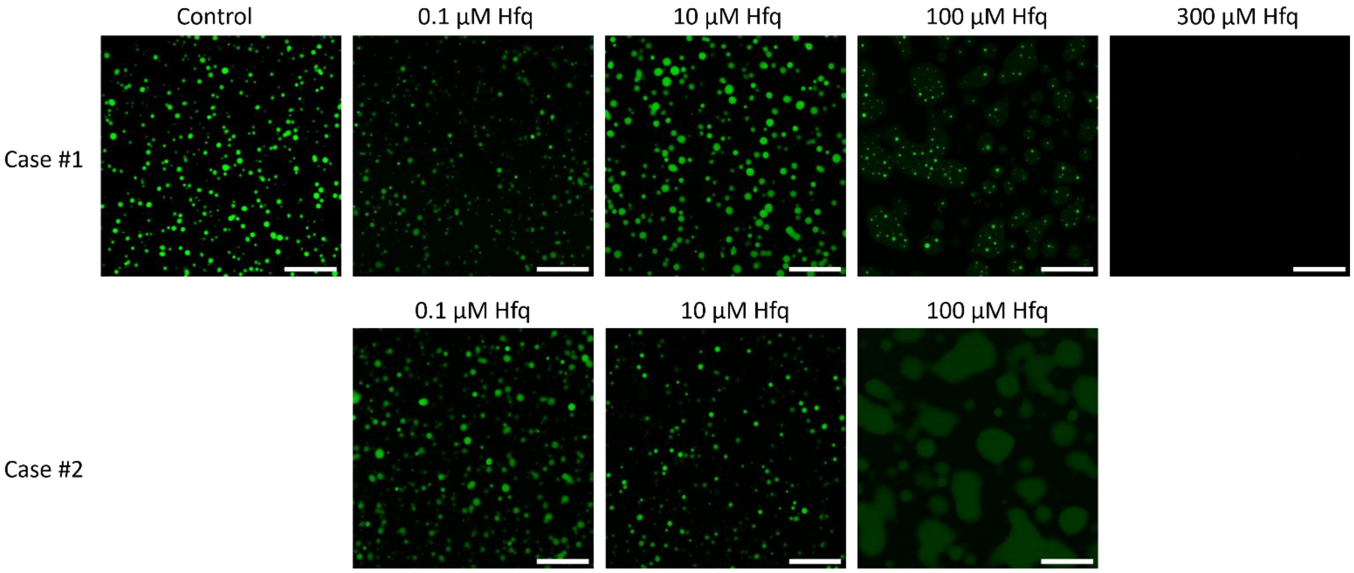

Supplementary Figure 19. Buforin-2 vs. Hfq competition assay. Case #1: 300 µM Buforin-2 was mixed with 250 ng/µL of poly(A) and incubated for 5 min at room temperature. Subsequently, 0.1-300 µM Hfq was introduced and the sample was incubated for an additional 5 min before imaging via a confocal fluorescence microscope. Case #2: 0.1-100 µM Hfq was pre-incubated with 250 ng/µL of poly(A) for 5 min before introducing 300 µM Buforin-2. Experiments were repeated 3 times with similar results. The scale bars are 20 µm.

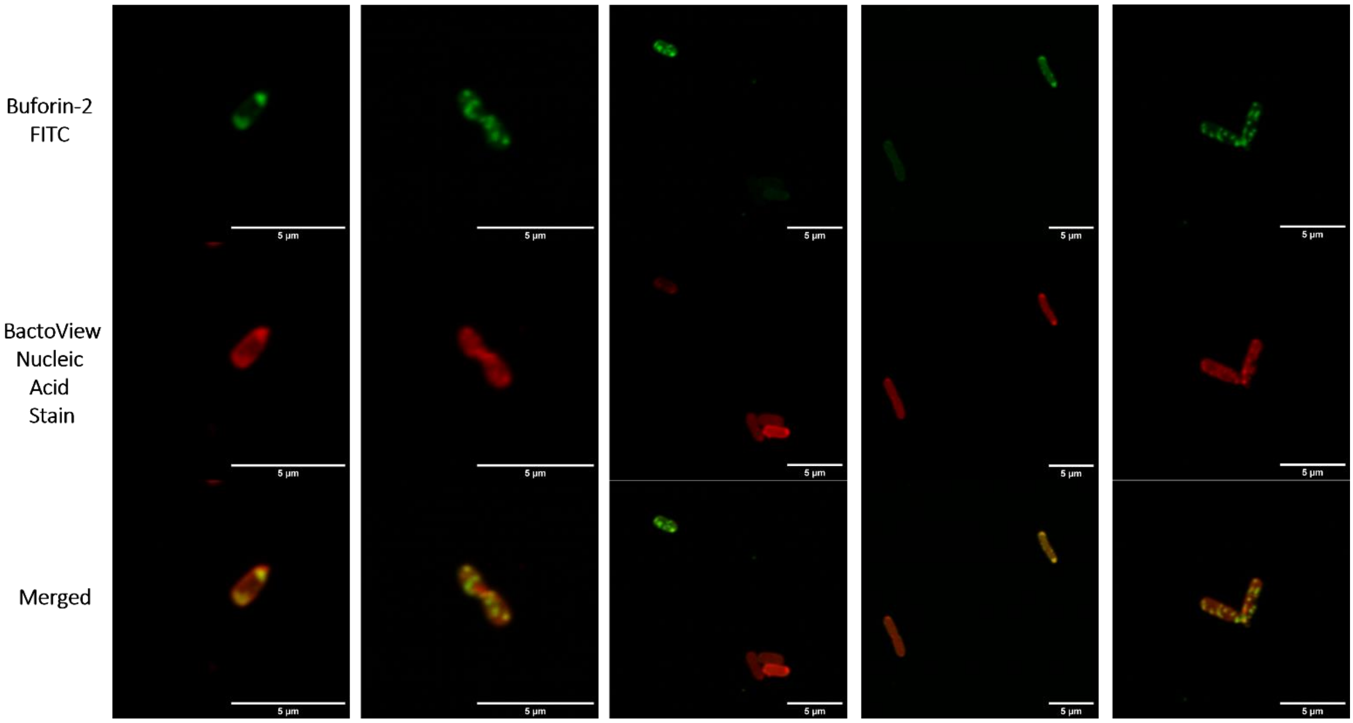

Supplementary Figure 20. Confocal microscopy images of E. coli cells incubated with 20 µM of FITC-Buforin-2 (green) for 2 h. Nucleic acids stained with BactoView Red (red). Experiments were repeated 2 times with similar results.

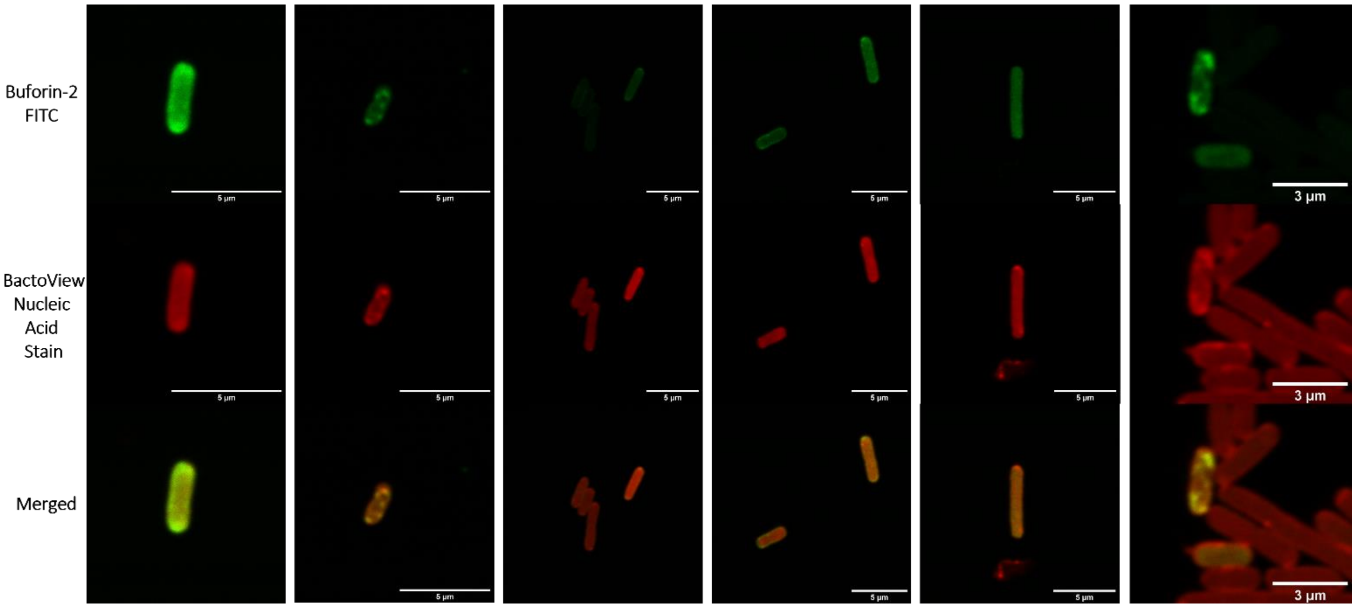

Supplementary Figure 21. Confocal microscopy images of E. coli cells incubated with 20 µM of FITC-Buforin-2 (green) for 2 h. Nucleic acids stained with BactoView Red (red). Experiments were repeated 2 times with similar results.

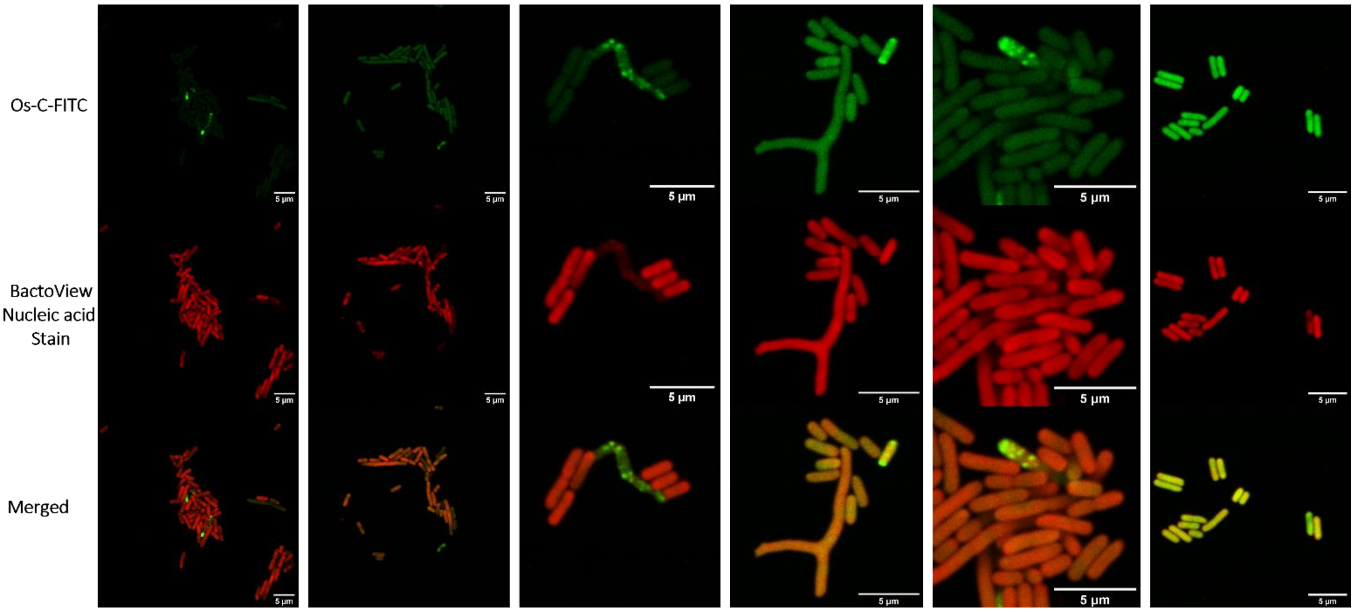

Supplementary Figure 22. Confocal microscopy images of E. coli cells incubated with 20 μM of FITC-Os-C (green) for 2 h. Nucleic acids stained with BactoView Red (red). Experiments were repeated 2 times with similar results.

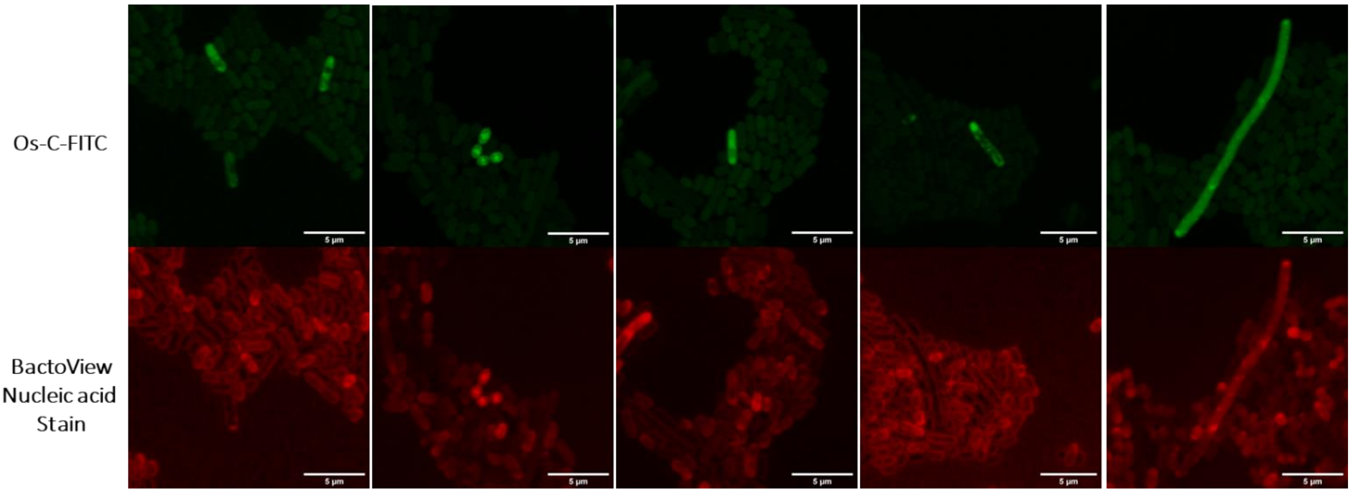

Supplementary Figure 23. Confocal microscopy images of E. coli cells incubated with 20 μM of FITC-Os-C (green) for 2 h. Nucleic acids stained with BactoView Red (red). Experiments were repeated 2 times with similar results.

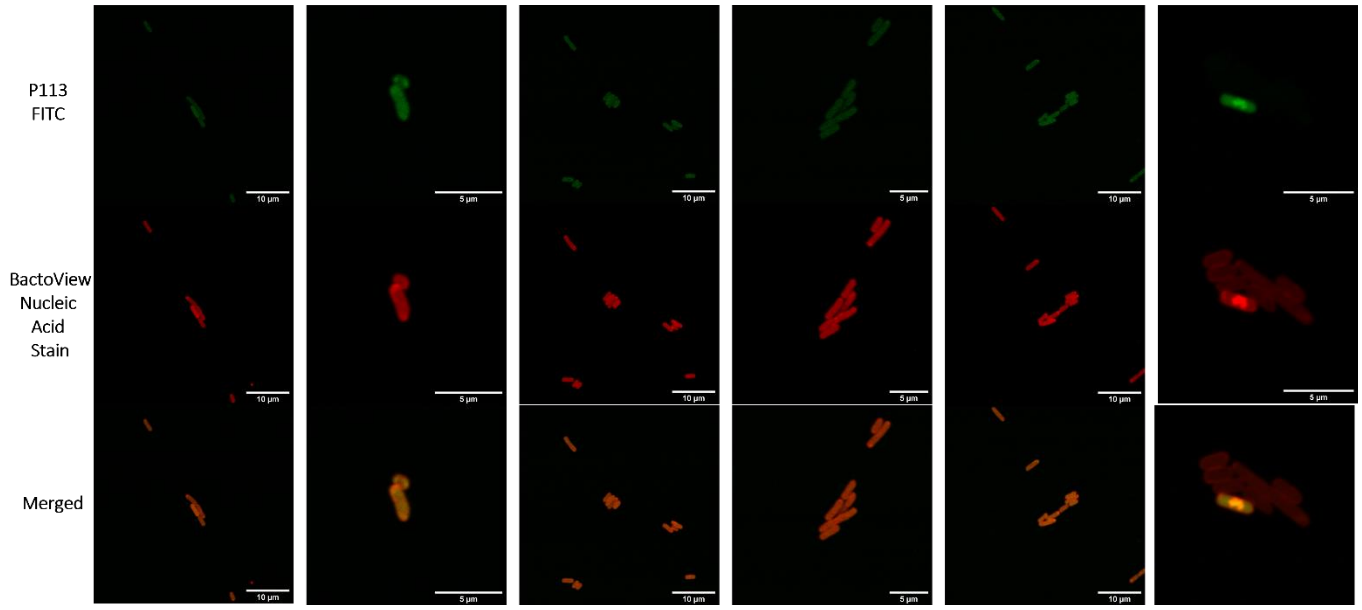

Supplementary Figure 24. Confocal microscopy images of E. coli cells incubated with 20 µM of FITC-P113 (green) for 2 h. Nucleic acids stained with BactoView Red (red). Experiments were repeated 2 times with similar results.

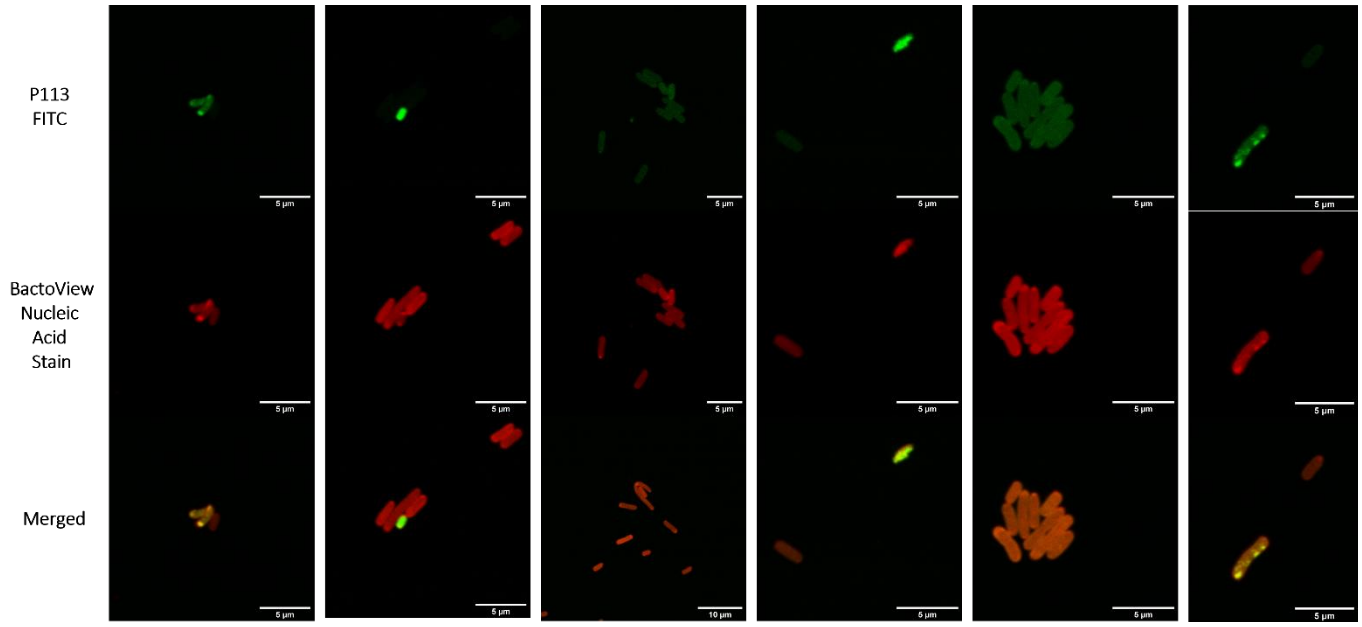

Supplementary Figure 25. Confocal microscopy images of E. coli cells incubated with 20 µM of FITC-P113 (green) for 2 h. Nucleic acids stained with BactoView Red (red). Experiments were repeated 2 times with similar results.

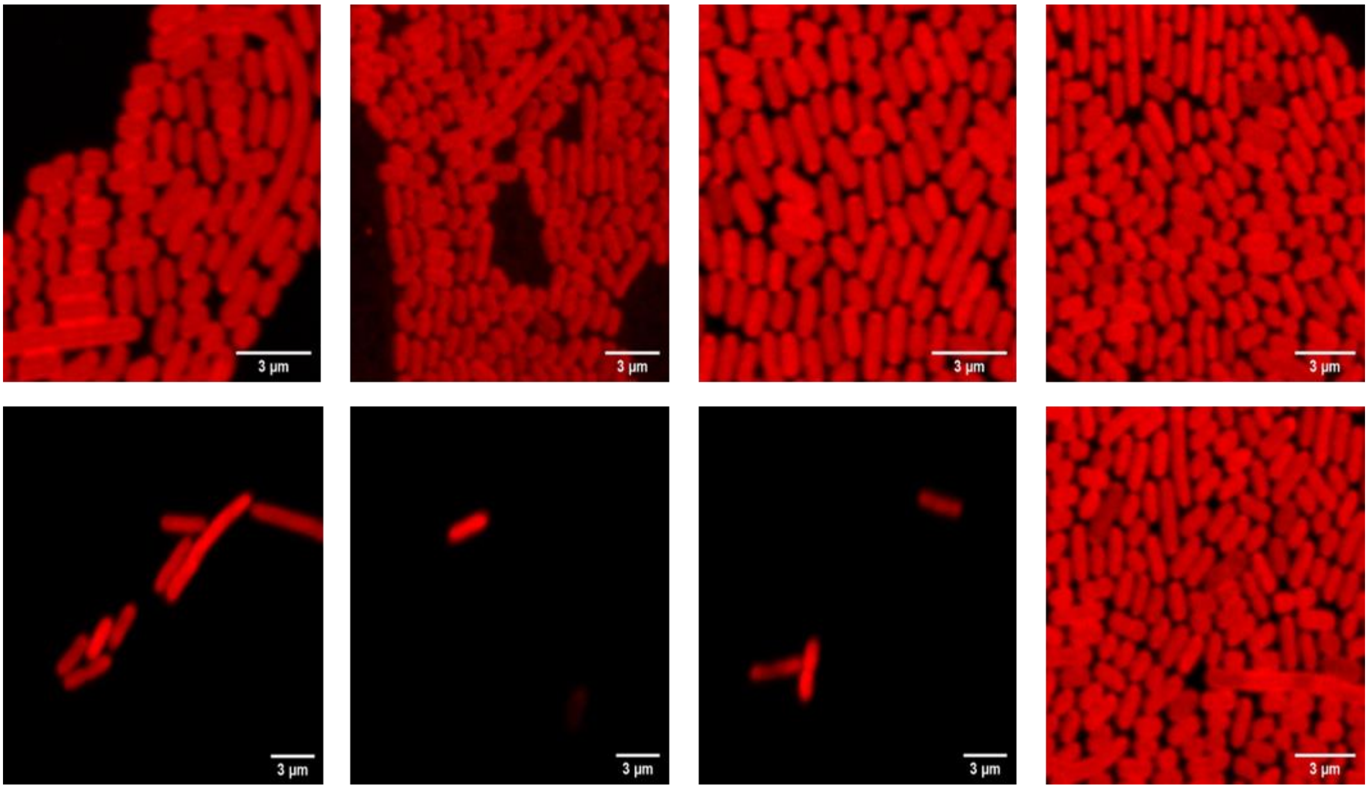

Supplementary Figure 26. Confocal microscopy images of E. coli cells incubated for 2 h without AMP present. Nucleic acids stained with BactoView Red (red). Scale bars are 3 μm. Experiments were repeated 2 times with similar results.

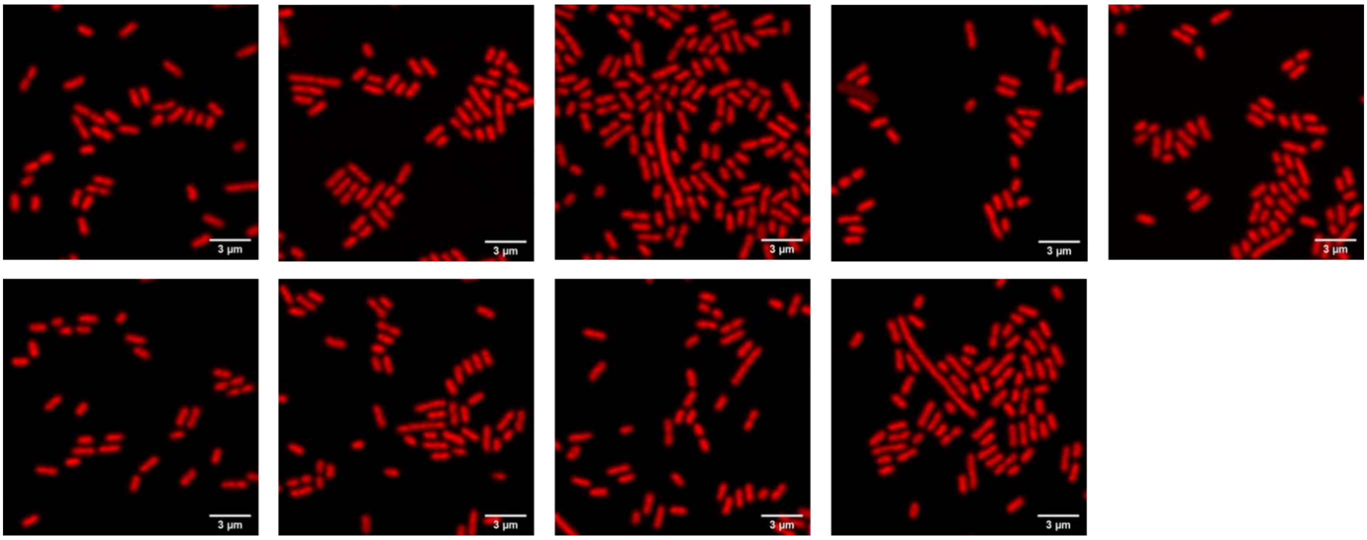

Supplementary Figure 27. Confocal microscopy images of E. coli cells incubated for 2 h without AMP present. Nucleic acids stained with BactoView Red (red). Scale bars are 3 μm. Experiments were repeated 2 times with similar results.

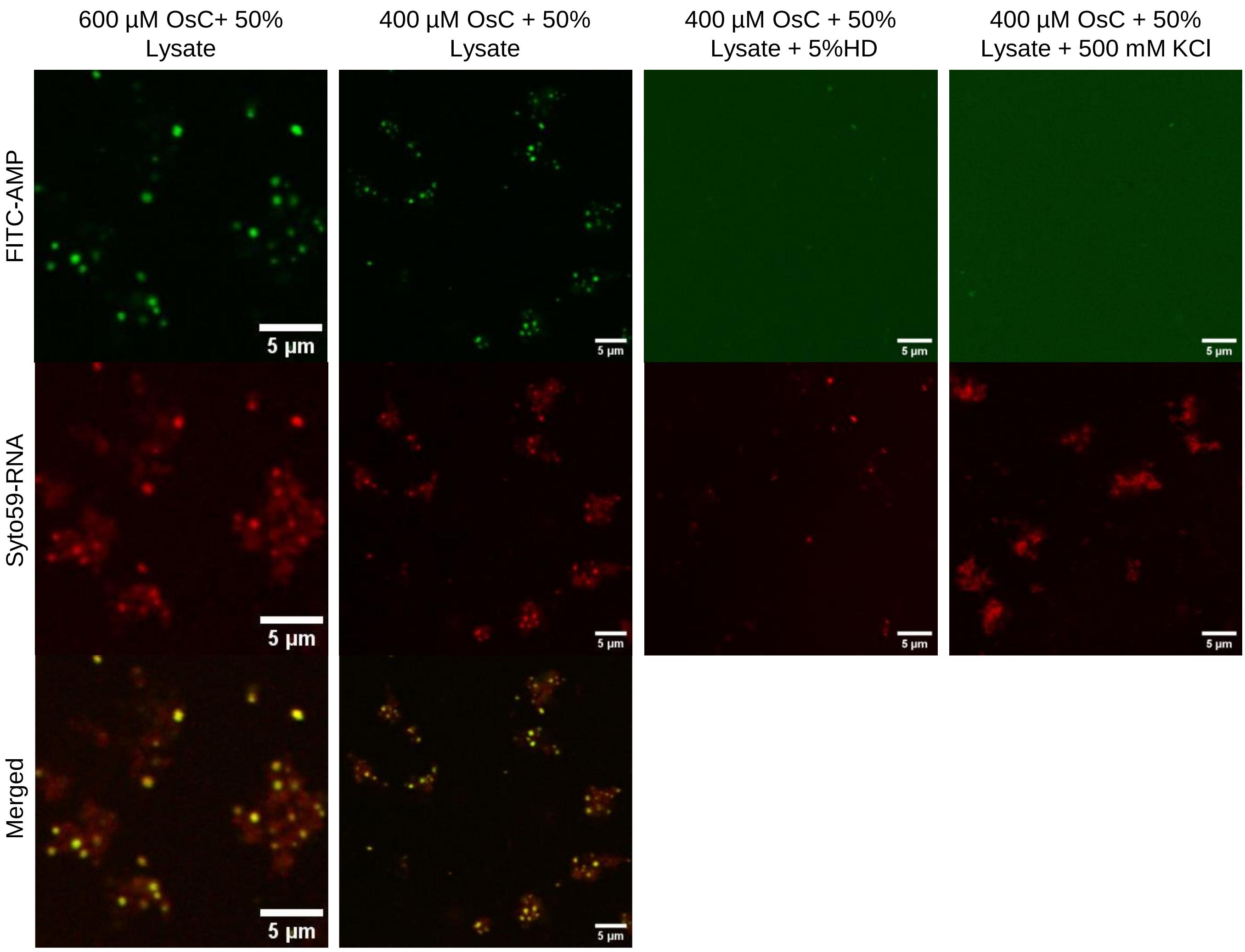

Supplementary Figure 28. Os-C induce liquid-like condensate formation inside extract-based active transcription/translation system derived from multiple E. coli cells. Experiments were repeated 3 times with similar results.

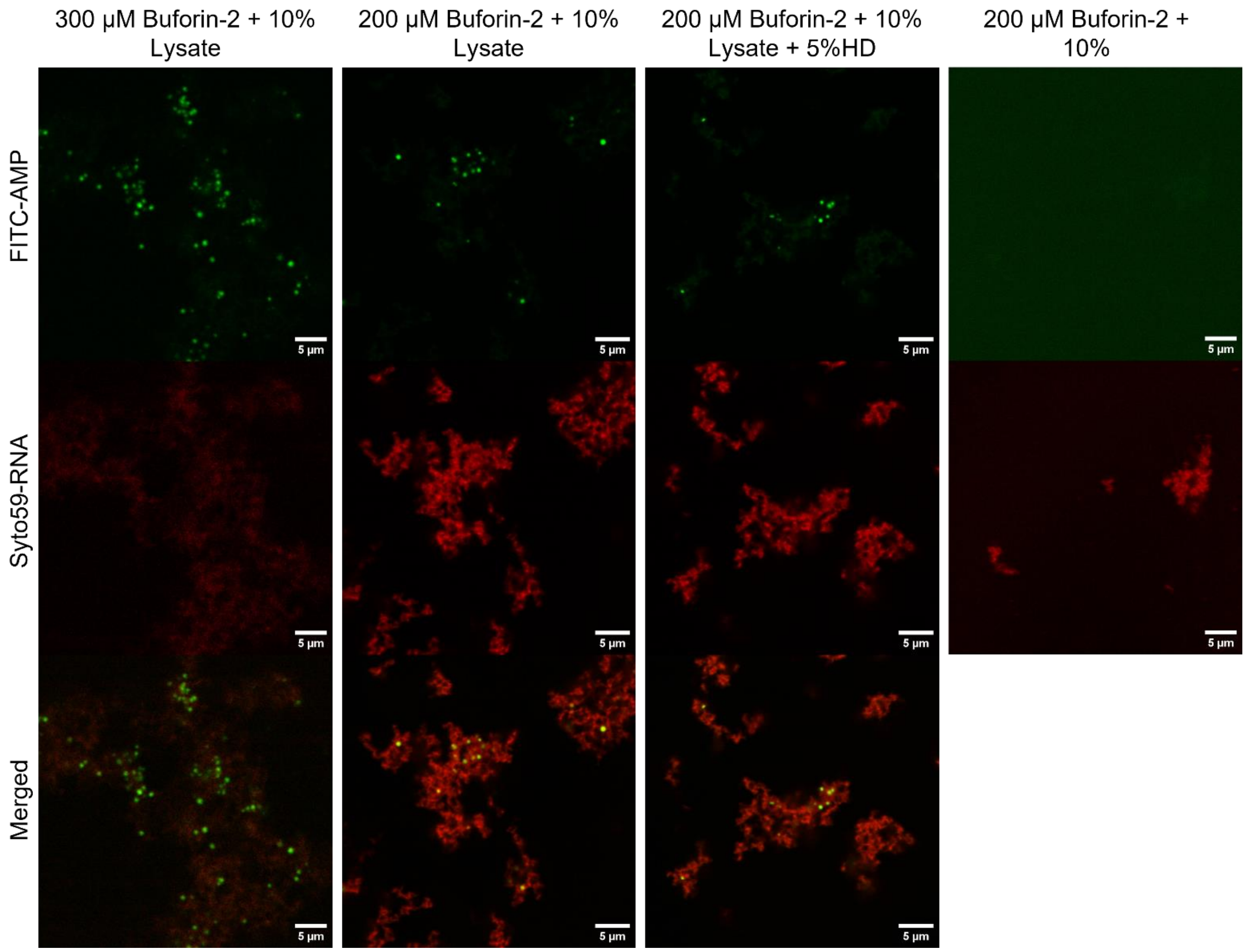

Supplementary Figure 29. Buforin-2 induce liquid-like condensate formation inside extract-based active transcription/translation system derived from multiple *E. coli* cells. Experiments were repeated 3 times with similar results.

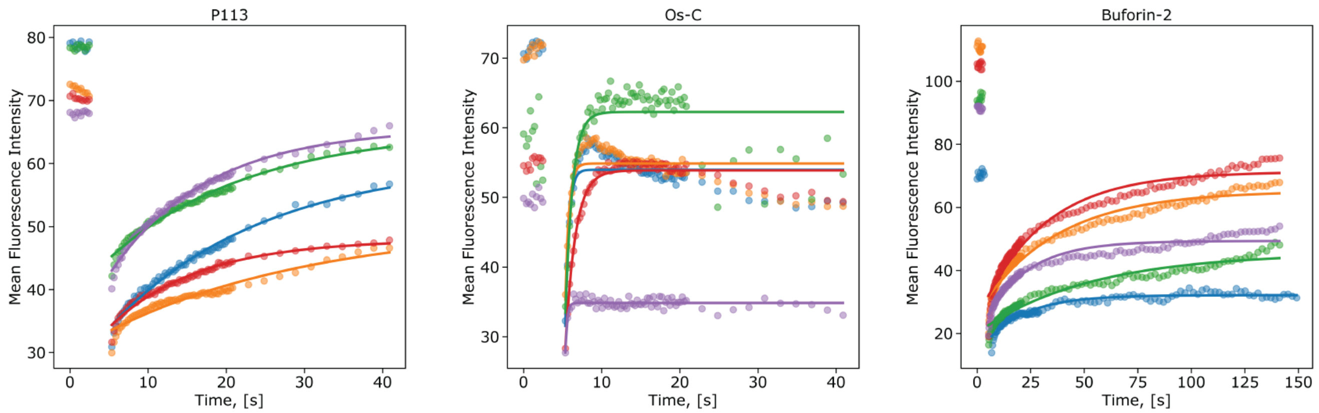

Supplementary Figure 30. Kymographs of the FRAP experiments performed with condensates formed in *E. coli* lysate (n = 5).
