# Supplementary material for: Targeting nucleic acid phase transitions as a mechanism of action for antimicrobial peptides
Source: Nat Commun. 2023 Nov 7;14:7170. doi: 10.1038/s41467-023-42374-4 (PMC10630377; doi:10.1038/s41467-023-42374-4)
Supplement: Supplementary file 3 — Description of Additional Supplementary Files [file 41467_2023_42374_MOESM3_ESM.pdf]

<u>Description of Additional Supplementary Files</u>

Supplementary Movie 1: P113-poly(A) RNA condensate fusion events.

Supplementary Movie 2: OsC-poly(A) RNA condensate fusion events.

Supplementary Movie 3: Buforin-2-poly(A) RNA condensate fusion events.
