# Supplementary material for: Targeting nucleic acid phase transitions as a mechanism of action for antimicrobial peptides
Source: Nat Commun. 2023 Nov 7;14:7170. doi: 10.1038/s41467-023-42374-4 (PMC10630377; doi:10.1038/s41467-023-42374-4)
Supplement: Supplementary file 7 — Reporting Summary [file 41467_2023_42374_MOESM7_ESM.pdf]

nature portfolio

Corresponding author(s): Prof. Tuomas P. J. Knowles
Dr Alexander Borodavka

Last updated by author(s): Sep 19, 2023

# Reporting Summary

Nature Portfolio wishes to improve the reproducibility of the work that we publish. This form provides structure for consistency and transparency in reporting. For further information on Nature Portfolio policies, see our Editorial Policies and the Editorial Policy Checklist.

## Statistics

For all statistical analyses, confirm that the following items are present in the figure legend, table legend, main text, or Methods section.

| n/a | Confirmed | |
|---|---|---|
| ☐ | ☒ | The exact sample size (*n*) for each experimental group/condition, given as a discrete number and unit of measurement |
| ☐ | ☒ | A statement on whether measurements were taken from distinct samples or whether the same sample was measured repeatedly |
| ☒ | ☐ | The statistical test(s) used AND whether they are one- or two-sided<br>*Only common tests should be described solely by name; describe more complex techniques in the Methods section.* |
| ☒ | ☐ | A description of all covariates tested |
| ☐ | ☒ | A description of any assumptions or corrections, such as tests of normality and adjustment for multiple comparisons |
| ☐ | ☒ | A full description of the statistical parameters including central tendency (e.g. means) or other basic estimates (e.g. regression coefficient) AND variation (e.g. standard deviation) or associated estimates of uncertainty (e.g. confidence intervals) |
| ☒ | ☐ | For null hypothesis testing, the test statistic (e.g. *F*, *t*, *r*) with confidence intervals, effect sizes, degrees of freedom and *P* value noted<br>*Give P values as exact values whenever suitable.* |
| ☒ | ☐ | For Bayesian analysis, information on the choice of priors and Markov chain Monte Carlo settings |
| ☒ | ☐ | For hierarchical and complex designs, identification of the appropriate level for tests and full reporting of outcomes |
| ☒ | ☐ | Estimates of effect sizes (e.g. Cohen's *d*, Pearson's *r*), indicating how they were calculated |

*Our web collection on statistics for biologists contains articles on many of the points above.*

## Software and code

Policy information about availability of computer code

| | |
|---|---|
| Data collection | Las X, inbuilt Leica Stellaris 5 software; Micro-Manager 2.0.0; inbuilt Fluidity one-M software; inbuilt FLUOstar Omega software; Database of Antimicrobial Activity and Structure of Peptides: https://dbaasp.org/search; APD3 ANTIMICROBIAL PEPTIDE DATABASE: https://aps.unmc.edu/database/anti; |
| Data analysis | Python 3.7; Spyder 5.2.2; Microsoft Excel 365; Fiji Image J 1.53t; MATLAB R2021b; Python-based version of DeePhase v1, including its modified version: https://github.com/kadiliissaar/deephase; Python-based combinatorial microdroplet analysis script (PhaseScan): https://github.com/rqi14/PhaseScan; Python-based script for calculation of tie-line gradients: chrome-extension://efaidnbmnnnibpcajpcglclefindmkaj/https://journals.aps.org/prx/pdf/10.1103/PhysRevX.12.041038; |

For manuscripts utilizing custom algorithms or software that are central to the research but not yet described in published literature, software must be made available to editors and reviewers. We strongly encourage code deposition in a community repository (e.g. GitHub). See the Nature Portfolio guidelines for submitting code & software for further information.

# Data

Policy information about availability of data

All manuscripts must include a data availability statement. This statement should provide the following information, where applicable:

- Accession codes, unique identifiers, or web links for publicly available datasets
- A description of any restrictions on data availability
- For clinical datasets or third party data, please ensure that the statement adheres to our policy

All data generated or analyzed during this study are included in this published article and its supplementary information files. Source data are provided with this paper.

# Human research participants

Policy information about studies involving human research participants and Sex and Gender in Research.

| | |
|---|---|
| Reporting on sex and gender | This information has not been collected. |
| Population characteristics | N/A |
| Recruitment | N/A |
| Ethics oversight | N/A |

Note that full information on the approval of the study protocol must also be provided in the manuscript.

# Field-specific reporting

Please select the one below that is the best fit for your research. If you are not sure, read the appropriate sections before making your selection.

☒ Life sciences ☐ Behavioural & social sciences ☐ Ecological, evolutionary & environmental sciences

For a reference copy of the document with all sections, see nature.com/documents/nr-reporting-summary-flat.pdf

# Life sciences study design

All studies must disclose on these points even when the disclosure is negative.

**Sample size**

No sample-size calculation was performed. Samples were chosen based on the results of DeePhase amino acid sequence analysis and previously published observations:
[1] Taute, H., Bester, M. J., Neitz, A. W. & Gaspar, A. R. Investigation into the mechanism of action of the antimicrobial peptides Os and Os-C derived from a tick defensin. Peptides 71, 179–187. issn: 18735169 (2015).
[2] Li, J. et al. Anti-infection peptidomics of amphibian skin. Molecular and Cellular Proteomics 6,882–894. issn: 15359476 (2007).
[3] Kozlowska, J. et al. Combined Systems Approaches Reveal Highly Plastic Responses to Antimicrobial Peptide Challenge in Escherichia coli. PLoS Pathogens 10. issn: 15537374 (2014).
[4] Cho, J. H., Sung, B. H. & Kim, S. C. Buforins: Histone H2A-derived antimicrobial peptides from toad stomach. Biochimica et Biophysica Acta - Biomembranes 1788, 1564–1569. issn: 00052736698 (2009).
[5] Park, C. B., Kim, H. S. & Kim, S. C. Mechanism of action of the antimicrobial peptide buforin II: Buforin II kills microorganisms by penetrating the cell membrane and inhibiting cellular functions. Biochemical and Biophysical Research Communications 244, 253–257. issn: 0006291X702 (1998).
[6] Uyterhoeven, E. T., Butler, C. H., Ko, D. & Elmore, D. E. Investigating the nucleic acid interactions and antimicrobial mechanism of buforin II. FEBS Letters 582, 1715–1718. issn: 00145793705 (2008).
[7] Muñoz-Camargo, C. et al. Unveiling the multifaceted mechanisms of antibacterial activity of buforin II and frenatin 2.3S peptides from skin micro-organs of the orinoco lime treefrog (Sphaenorhynchus lacteus). International Journal of Molecular Sciences 19. issn: 14220067709 (2018).

| | |
|---|---|
| Data exclusions | No data was excluded from the analyses. |
| Replication | Where possible, experiments were replicated at least three times to ensure reproducibility. |
| Randomization | Not relevant to this study, no samples/organisms/participatns were allocated into experimental groups. |
| Blinding | Not relevant to his study, no group allocation was performed during data collection and/or analysis. |

# Reporting for specific materials, systems and methods

We require information from authors about some types of materials, experimental systems and methods used in many studies. Here, indicate whether each material, system or method listed is relevant to your study. If you are not sure if a list item applies to your research, read the appropriate section before selecting a response.

## Materials & experimental systems

| n/a | Involved in the study |
| --- | --- |
| ☒ | ☐ Antibodies |
| ☒ | ☐ Eukaryotic cell lines |
| ☒ | ☐ Palaeontology and archaeology |
| ☒ | ☐ Animals and other organisms |
| ☒ | ☐ Clinical data |
| ☒ | ☐ Dual use research of concern |

## Methods

| n/a | Involved in the study |
| --- | --- |
| ☒ | ☐ ChIP-seq |
| ☒ | ☐ Flow cytometry |
| ☒ | ☐ MRI-based neuroimaging |
